# Coordinated Multi-Robotic Vehicles Navigation and Control in Shop Floor Automation

**DOI:** 10.3390/s22041455

**Published:** 2022-02-14

**Authors:** Gregor Klančar, Marija Seder

**Affiliations:** 1 Faculty of Electrical Engineering, University of Ljubljana, Tržaška 25, SI-1000 Ljubljana, Slovenia; 2 Faculty of Electrical Engineering and Computing, University of Zagreb, Unska 3, 10000 Zagreb, Croatia; marija.seder@fer.hr

**Keywords:** navigation, model predictive control, path planing, mobile robots, warehouse automation

## Abstract

In this paper, we propose a global navigation function applied to model predictive control (MPC) for autonomous mobile robots, with application to warehouse automation. The approach considers static and dynamic obstacles and generates smooth, collision-free trajectories. The navigation function is based on a potential field derived from an E* graph search algorithm on a discrete occupancy grid and by bicubic interpolation. It has convergent behavior from anywhere to the target and is computed in advance to increase computational efficiency. The novel optimization strategy used in MPC combines a discrete set of velocity candidates with randomly perturbed candidates from particle swarm optimization. Adaptive horizon length is used to improve performance. The efficiency of the proposed approaches is validated using simulations and experimental results.

## 1. Introduction

Mobile robotic platforms have found numerous applications over the past decade, a significant portion of which can be attributed to intralogistics and transportation in modern manufacturing, warehousing, and utility markets [1,2,3]. Although numerous sites have been automated by either automated guided vehicles (AGV) or autonomous mobile robots (AMR) with impressive deployments, the market is expected to grow by about 30% over the next five years [4,5].

Since the first AGV was built in 1953 [3], AGVs have evolved into today’s solution, which is the standard in automating internal logistics. Typically, AGVs move along predefined paths and can only deliver to fixed points along the path. This makes these transportation systems simpler and more robust. Since movement is limited to fixed paths, the complexity of path planning and coordinating multiple AGVs is reduced. Nevertheless, planning collision-proof safe paths for a group of AGVs and creating and optimizing schedules to achieve better performance (higher throughput and less likely occurrence of conflicts) remains a challenging task [6]. Path planning is usually solved using graph-based search algorithms such as A*-based search, where optimal approaches [7,8] are feasible for a smaller number of vehicles since the computational complexity is exponential with the number of vehicles. The coordination overhead in multi-AGV systems is further reduced by suboptimal approaches, where the problem is decoupled from finding individual vehicles and conflicts are resolved by assigning traffic rules, priorities, or distributed multi-agent negotiations [6,9,10].

AMRs (unlike AGVs) are more flexible (in terms of their navigation capabilities and the services they can provide) and can move freely in dynamic environments where they locate, navigate, and act autonomously [4]. Free space is mapped based on knowledge of static obstacles, and dynamic obstacles are avoided using sensors. Since movement is not restricted to predefined paths but is possible in the continuum of obstacle-free space, the complexity of path planning must be reduced. A common approach is to discretize the environment into cells of equal size and use grid-based path planning [11,12]. Since pathfinding usually examines only 4 or 8 neighbourhood directions, the paths obtained are not smooth.

Another approach is to apply a discrete set of motion primitives or actions that a vehicle can apply to advance to new locations. The motion primitives can be Bezier curves [13,14], clothoids [15], or other smooth curvature curves [16,17,18]. This usually results in smoother paths that a vehicle can easily follow. Different optimization strategies can be used to select suitable motion primitives. In high-dimensional spaces, randomised planners such as the Rapid Exploring Random Tree (RRT*) and the kinodynamic RRT* are popular choices [3,19]. A state lattice graph can be constructed from a discrete set of motion primitives that have smooth curvature transitions in the joints [20,21]. Graph search algorithms such as A* [22,23], D* [11,24], and E* [25] can be used to find the final path. The computational complexity can be addressed by hybrid search approaches such as Hybrid A* or HE * [14,26], where a computationally efficient discrete graph-based search is applied to obtain the heuristics for more efficient construction and search of the lattice graph, where the motion primitives form the edges of the graph.

Potential field-planning methods are also popular, where the potential function for online navigation can be used to guide the search or control algorithm. The goal with minimum potential value can be achieved by simply following the direction of the steepest descent of the potential field. A common problem of the potential field is local minima in which the robot may be trapped. Several approaches have been proposed to avoid local minima. Concave obstacles can be simply modelled as convex [27] in the environment map, or an adaptive potential field can be generated using multiple points of attraction instead of just one in the target [28]. It is also possible to modify the potential field in unstable equilibrium by introducing perturbations into the field [29] or adding virtual obstacles to repel the robot from the local minima [30]. The potential field can also be interpolated from a discrete cost map obtained from an optimal grid-based search [31,32]. Relying only on the reactive behavior of a potential field may result in unwanted oscillations in the presence of obstacles where alternating repulsive and attractive fields may cause approaching and moving-away behavior [33]. Therefore, prediction capabilities are needed to achieve more deliberative actions where planning and control are combined in receding dynamic window approaches or trajectory roll-out algorithms considering convergent navigation function, such as in [31,34,35,36]. Here, the obtained performance depends on a control law and uses an objective function, which needs to incorporate mapped static obstacles and sensed dynamic obstacles to find feasible optimal trajectories in a prediction horizon.

Moving obstacle avoidance is required for efficient multiple vehicle navigation. Coordinated motion of multiple vehicles can be dealled by assigning traffic rules in decentralised manner as in [10] or by combining a centralized supervisor, which detects collisions and assignes priorities for decentralised planner and scheduling for collision avoidance [37]. The decentralised decoupled approach is proposed in [18], where vehicles first plan optimal paths independently; then, conflict resolution is performed based on a priority scheme. In [38], a model predictive scheme is proposed where local deviations from the existing reference path are optimized considering collision avoidance with static and moving obstacles. In [39], an integration of the focused D* graph search algorithm for path planning and the dynamic window algorithm for generating admissible robot trajectories around the planned global path is proposed.

In this paper, the main contributions are the following. We propose a global navigation function applied in model predictive control to safely navigate the vehicle to the goal destination. The navigation function depends on a potential field for the environment layout and the driving direction. The potential function for a known target is computed in advance by an E* graph search algorithm on a discrete rectangular grid. A smooth surface with arbitrary potential values and slope directions is obtained by bicubic interpolation. It allows navigation from any location to the target and can be precomputed for any known target to which AMR must deliver.

Constrained Model Predictive Control (MPC) is a method of finding optimal trajectories given the proposed navigation function and constraints on robot kinematics, maximum velocities and accelerations, convergent behavior, and the coordination of multiple robots. MPC combines local motion planning and control in the presence of static and dynamic obstacles.

Adaptive horizon length is introduced in MPC to improve performance in terms of safety and achieved curve optimality.

A novel optimization strategy for MPC is proposed that combines a discrete set of command velocities proposed in [36] with randomly perturbed particle swarm optimization candidates. This approach extends the navigation of a single robot [32] to multiple robots.

Coordinated navigation in the presence of multiple vehicles is obtained locally as a constraint in the MPC objective function. The approach assumes that cooperative vehicles share their planned trajectories within the prediction horizon. For non-cooperative objects, the motion trajectories must be estimated from measurements.

The performance of the proposed approaches is illustrated by several examples.

## 2. Vehicle Autonomous Navigation and Control

**Figure 1:**
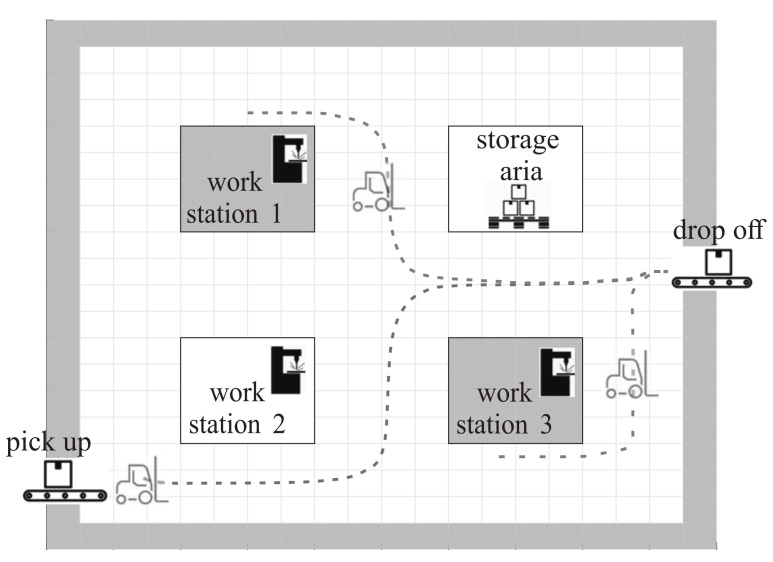
Production layout with multiple known and fixed delivery points and defined free corridors. Shown are robot delivery paths from three starting locations to the same destination. The paths can be effectively determined by a single navigation function, as shown in Figure 6.

For an example of a simple production layout, see Figure 1, where robots typically need to transport material between known, fixed locations. Suppose a destination is the dropoff point shown in Figure 1, to which robots must deliver material from several other locations, such as pickup point, workstations, and storage aria. A navigation function can be created to guide the robot safely from any starting point in the environment to the desired destination.

**Figure 2:**
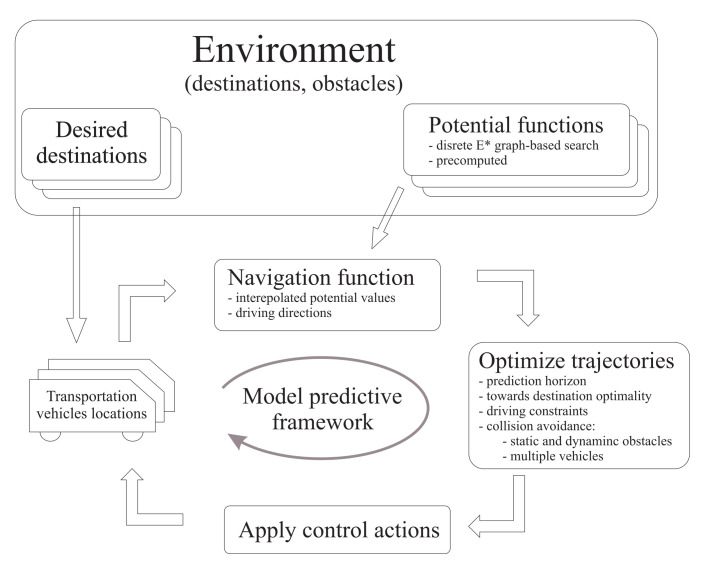
Basic idea of navigation and control of multiple vehicles based on the proposed navigation function and model predictive control.

Basic idea of applied navigation and control diagram is illustrated in Figure 2.

**Figure 3:**
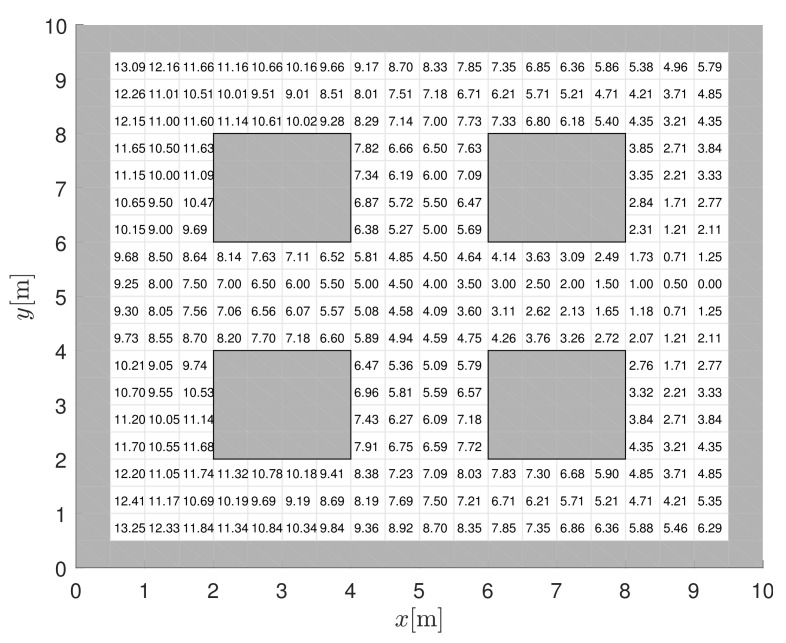
3D view of the discrete potential function from obtained a grid-based search with 0.5 m resolution, a target position at x=9.25 m, y=5.25 m, and occupied cells with U(x,y)=∞ (grey cells).

In Figure 1, three paths are shown that are automatically determined based on the derived navigation function shown in Figure 6, which is interpolated at runtime from a stored discrete potential field of the layout, as shown in Figure 3. Other navigation functions are determined for other desired frequent destinations such as pickup belts, workstations, or battery charging arias. Such navigation functions can be computed in advance if the pickup and drop-off locations are fixed and the robots can move in predefined corridors. This leads to a computationally efficient approach with high-quality trajectories that takes static obstacles into account during design and can also be extended to include detected dynamic obstacles. Further details of the navigation function and control algorithm are presented below.

### 2.1. Concept of Navigation

The navigation function N(x,y,φ) is used to drive a wheeled robot with position *x*, *y* and orientation φ) safely between obstacles to the goal. A control algorithm therefore steers the robot to locations where N(x,y,φ) decreases. The unimodal potential function is a good choice for N(x,y,φ) because it has a single minimum (N(x,y,φ)=0) at the goal and no local minima where the control algorithm might get stuck. Additionally, N(x,y,φ) must have the highest values at the obstacles. A graph search algorithm such as D${}^{*}$ for dynamic environments can be used to obtain such a potential function U(x,y) as shown in Figure 3. The value of U(x,y) represents the distance to the target cell, which is computed as the sum of the distances between cells ($d_{c}$) along the path. Such a search is computationally efficient since it is performed on a discrete grid of the environment but is not suitable for a control algorithm since U(x,y) is constant for any robot position within a discrete cell. Therefore, the grid-based navigation function must be modified to obtain a unique value for each position within a cell that retains the property of a single minimum [31]. In the following, we propose a bicubic interpolation approach.

### 2.2. Bicubic Interpolation

**Figure 4:**
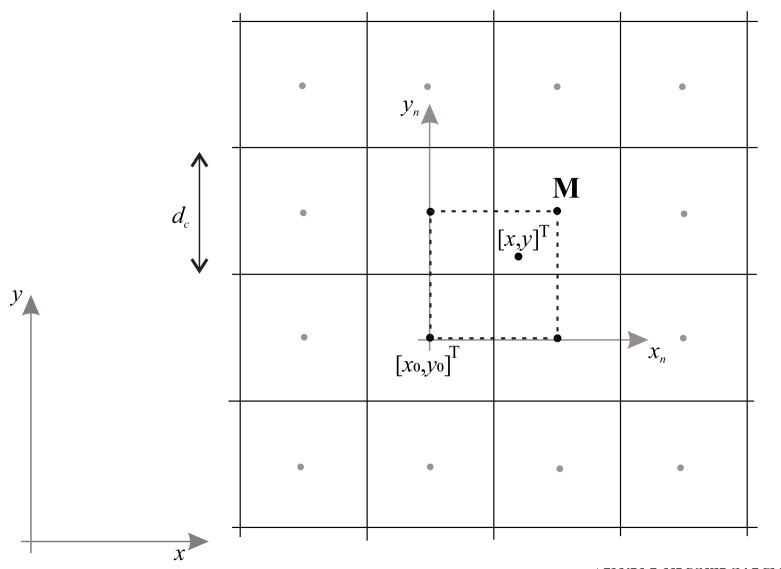
Selection of the cell neighbourhood for the bicubic interpolation of the potential field based on a point ${\left[x,y\right]}^{T}$ within the cell M.

To obtain a smooth interpolated potential P(x,y) from a discrete potential U(x,y), bicubic interpolation [40] is used. For a given arbitrary position ${\left[x,y\right]}^{T}$ within a cell M, an interpolated potential is calculated based on a 4 x 4 cell neighborhood, as shown in Figure 4. Depending on the quadrant of cell M in which the point ${\left[x,y\right]}^{T}$ is located, an appropriate four-cell neighborhood is determined whose centers form a square, as shown in Figure 4 shown by a dashed line.

The normalized coordinates $x_{n},y_{n}\in \left[0,1\right]$ are defined as $x_{n}=\frac{x-x_{0}}{d_{c}}$, $y_{n}=\frac{y-y_{0}}{d_{c}}$, where the origin $\left[x_{0},y_{0}\right]$ is defined by the lower left corner of the dashed square and $d_{c}$ is the cell size. The interpolated and discrete potential in normalized coordinates are expressed as $P_{n}\left(x_{n},y_{n}\right)=P\left(x,y\right)$ and $U_{n}\left(x_{n},y_{n}\right)=U\left(x,y\right)$, respectively. Define the potential and estimated partial derivatives for the four adjacent cell centers (corners of a dashed square in Figure 4)

$$p_{rc}=U_{n}\left(x_{n},y_{n}\right){|}_{x_{n}=r,\;y_{n}=c}$$

$$f_{xrc}=\frac{\partial P_{n}}{\partial x_{n}}{|}_{x_{n}=r,\;y_{n}=c}\approx \frac{U_{n}\left(r+1,c\right)-U_{n}\left(r-1,c\right)}{2}$$

$$f_{yrc}=\frac{\partial P_{n}}{\partial y_{n}}{|}_{x_{n}=r,\;y_{n}=c}\approx \frac{U_{n}\left(r,c+1\right)-U_{n}\left(r,c-1\right)}{2}$$

$$\begin{array}{c} f_{xyrc}=\frac{\partial^{2}P_{n}}{\partial x_{n}\partial y_{n}}{|}_{x_{n}=r,\;y_{n}=c}\approx \frac{U_{n}\left(r+1,c+1\right)-U_{n}\left(r-1,c+1\right)-U_{n}\left(r+1,c-1\right)-U_{n}\left(r-1,c-1\right)}{4}, \end{array}$$

where r,c∈{0,1} and $U_{n}\left(x_{n},y_{n}\right)=U\left(x,y\right)$.

For a given arbitrary position, the interpolated potential is then defined by bicubic interpolation as follows

(1) $$P_{n}\left(x_{n},y_{n}\right)=\left[1\;x_{n}\;x_{n}^{2}\;x_{n}^{3}\right]A{\left[1\;y_{n}\;y_{n}^{2}\;y_{n}^{3}\right]}^{T},$$

where the matrix of coefficients is

$$A=\left[\begin{array}{cccc} 1 & 0 & 0 & 0\\ 0 & 0 & 1 & 0\\ -3 & 3 & -2 & -1\\ 2 & -2 & 1 & 1 \end{array}\right]\left[\begin{array}{cccc} p_{00} & p_{01} & f_{y00} & f_{y00}\\ p_{10} & p_{11} & f_{y10} & f_{y11}\\ f_{x00} & f_{x01} & f_{xy00} & f_{xy01}\\ f_{x10} & f_{x11} & f_{xy10} & f_{xy11} \end{array}\right]\left[\begin{array}{cccc} 1 & 0 & -3 & 2\\ 0 & 0 & 3 & -2\\ 0 & 1 & -2 & 1\\ 0 & 0 & -1 & 1 \end{array}\right].$$

And negative gradient of P(x,y) in ${\left[x,y\right]}^{T}$ is computed in a closed-form as

(2) $$\begin{array}{ccc} \vec{g}\left(x,y\right) & = & -\nabla P\left(x,y\right)=-{\left[\frac{\partial P(x,y)}{\partial x},\frac{\partial P(x,y)}{\partial y}\right]}^{T}=\\ \; & = & -\frac{1}{d_{c}}{\left[\frac{\partial P(x_{n},y_{n})}{\partial x_{n}},\frac{\partial P(x_{n},y_{n})}{\partial y_{n}}\right]}^{T} \end{array}.$$

For non-holonomic robots (with the kinematics given in 6), the final navigation function depends on the interpolated potential P(x,y) and on the robot orientation φ

(3) $$\begin{array}{ccc} N(x,y,\varphi ) & = & P(x,y)+\xi e(\varphi )\\ e(\varphi ) & = & \min_{k=\{0,1,-1\}}\left|∠\vec{g}\left(x,y\right)-\varphi +2k\pi \right|, \end{array}$$

where e(φ) is the absolute orientation error, ξ>0, and $∠\vec{g}\left(x,y\right)$ is the orientation of the negative gradient.

**Figure 5:**
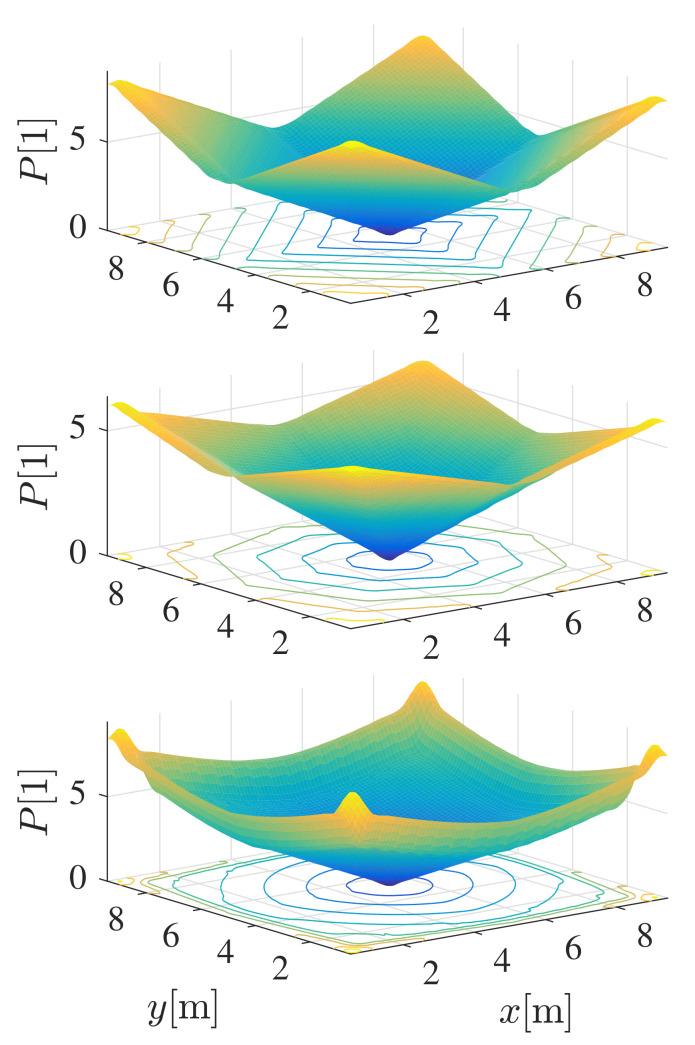
Interpolated potential function P(x,y) based on the discrete potential obtained by A${}^{*}$ with 4 and 8 neighbour cells connections and by E${}^{*}$. The obtained gradient is in the directions of multiples of 90 ${}^{\circ }$ or 45 ${}^{\circ }$ when 4 or 8 neighbourhood connections are used in A${}^{*}$. While E${}^{*}$ can have arbitrary directions, which can be seen from the contours of equal potentials orthogonal to the gradient direction.

In Figure 5, the interpolated potential function P(x,y) of the free space and the centrally located target is shown for discrete potentials obtained by A${}^{*}$ and E${}^{*}$ grid-based searches. Since A${}^{*}$ uses 4 and 8 neighbourhood connections, respectively, the gradients of P(x,y) remain multiples of 90${}^{\circ }$ and 45${}^{\circ }$, respectively (see contours of the same potential in Figure 5). The E${}^{*}$ [25] is a dynamic path planning algorithm that can approximate continuous gradients (and contours). It uses 4 neighbour connectivity (such as A${}^{*}$ or D${}^{*}$), but instead of one, two parent nodes in orthogonal directions are used to get a better cost-to-goal estimate for each cell. Using the discrete potential field obtained from the E${}^{*}$ algorithm, the interpolated potential function (1) is smoother with arbitrary direction of the negative gradient (2).

**Figure 6:**
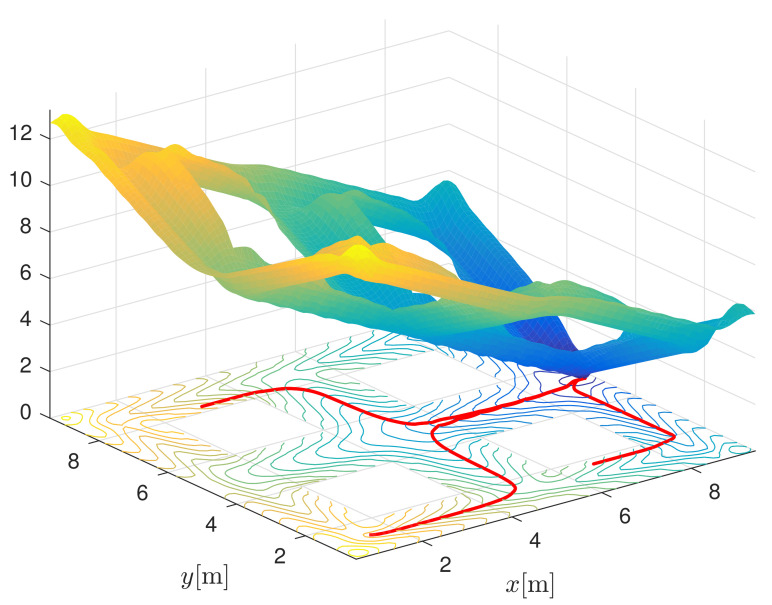
3D view of the interpolated navigation function N(x,y,φ). For clarity, e(φ) is set to zero in Equation (3). Three paths are drawn from different starting points, following the negative gradient towards the goal location with the lowest potential value.

An example of an occupied space and its computed interpolated smooth navigation function is given in Figure 6. Additionally, three paths are drawn from different starting points following the negative gradient towards the target with the lowest potential. The obtained paths are orthogonal to the contours of the same potential (see the lower part of Figure 6).

## 3. Coordinated Model Predictive Control

The proposed interpolated potential function Equation (1) allows the simple application of control of a single robot to safely navigate from anywhere to the target while automatically avoiding obstacles. The vehicle only needs to follow the given negative gradient direction Equation (2), and since the gradient has soft transitions (see e.g., Figure 3), feasible trajectories result. Such an approach lacks predictive capabilities and assumes a static environment without any other vehicles.

Therefore, the control behavior is defined as follows. The simple gradient-following reactive behavior is improved by incorporating prediction, so that the current control action also depends on the future states of the vehicles. The navigation function already includes knowledge of a static map in which the space occupied by obstacles has infinite potential. However, observed dynamic obstacles (cooperative obstacles such as other transport vehicles or non-cooperative obstacles such as humans or forklifts controlled by humans, etc.) are not included in the navigation function as this would require constant replanning. Dynamic obstacles are observed by sensors (laser range finder, camera, etc.), and their movement is estimated in the prediction horizon of the controller. A feasible trajectory is determined in the prediction horizon that is consistent with the navigation function, does not conflict with other vehicles or other detected obstacles, and is within the kinematic and dynamic constraints of the vehicle.

### 3.1. Control Definition

Model predictive control (MPC) is defined as an optimization problem with constraints. Optimal controls $u\left(t\right)={\left[v\left(t\right),\omega \left(t\right)\right]}^{T}$ are found for a differential robot over a prediction horizon *h* that minimize the objective function *J* at the current robot state $s\left(t\right)={\left[x,y,\varphi \right]}^{T}$

(4) $$\begin{array}{cc} J(s(t)) & =\min_{u(i-1)}\sum_{i=1}^{h}\left(N\left(s\left(i\right)\right)+u^{T}\left(i-1\right)Ru\left(i-1\right)\right)\\ \; & \mathrm{subject to}:\\ \; & -\;\mathrm{free configuration space}\;(\mathrm{Equation}\;(5))\\ \; & -\;\mathrm{driving constraints}\;(\mathrm{Equation}\;(7))\\ \; & -\;\mathrm{convergent behavior}\;(\mathrm{Equation}\;(11))\\ \; & -\;\mathrm{collision}-\mathrm{free with dynamic obstacles}\;(\mathrm{Equation}\;(12)) \end{array}$$

where *v* and ω are translational and angular velocity, *i* is a shorthand notation for time $t+iT_{s}$, $T_{s}$ is the sampling time, and R is the weighting matrix. Note that MPC considers the robot’s motion model and the environment model, where the target and static obstacles are already considered in the navigation function. However, due to kinematic constraints, collisions with static obstacles may still occur. Therefore, a valid trajectory in the horizon must lie in the free configuration space of the static map $Q_{free}$

(5) $$\begin{array}{ccc} s\left(i\right)\in Q_{free} & , & i=1,\ldots ,h. \end{array}$$

Similarly, collisions with dynamic obstacles are considered. For more details, see the Section 3.1.4. The future state of the robot $s\left(i\right)={\left[x\left(i\right),y\left(i\right),\varphi \left(i\right)\right]}^{T}$ is predicted using differential drive kinematics

(6) $$\begin{array}{c} x\left(i+1\right)=x\left(i\right)+v\left(i\right)T_{s}\cos\left(\varphi \left(i\right)+\frac{\omega \left(i\right)T_{s}}{2}\right)\\ y\left(i+1\right)=y\left(i\right)+v\left(i\right)T_{s}\sin\left(\varphi \left(i\right)+\frac{\omega \left(i\right)T_{s}}{2}\right)\\ \varphi \left(i+1\right)=\varphi \left(i\right)+\omega \left(i\right)T_{s}. \end{array}$$

#### 3.1.1. Driving Constraints

Control actions are constrained by maximum velocities and accelerations by

(7) $$\begin{array}{ccc} 0\leq v\left(i\right)\leq v_{\max} & , & \left|\omega \left(i\right)\right|\leq \omega_{\max}\\ \frac{\left|v(i)-v(i-1)\right|}{T_{s}}\leq a_{\max} & , & \frac{\left|\omega (i)-\omega (i-1)\right|}{T_{s}}\leq \alpha_{\max}, \end{array}$$

where $v_{\max}$, $\omega_{\max}$, $a_{\max}$, and $\alpha_{\max}$ are maximum allowable translational and rotational velocities and accelerations.

#### 3.1.2. Length of the Horizon

During the horizon, let the robot travel on an arc, where v(i)/ω(i) is its radius. A constant arc in the horizon is convenient because it reduces the computational cost of the MPC problem since it only requires the optimization of two parameters. The choice of horizon length affects the driving performance, safety, and computational cost of MPC.

The minimum horizon length ($h=h_{min}$) is chosen so that the robot travelling at maximum speed can safely decelerate to a stop at the end of the prediction horizon

(8) $$h_{min}=\left\lceil \max\left(\frac{v_{\max}}{a_{\max}T_{s}},\frac{w_{\max}}{\alpha_{\max}T_{s}}\right)\right\rceil +1.$$

This prevents the worst case collision with the maximum robot speed and the newly observed (static) object at the end of the prediction horizon. The control u(i), i∈0,1,…,h−1 in the horizon therefore decreases linearly to zero at the end of the horizon, as follows

(9) $$u\left(i\right)=\left\{\begin{array}{ccc} u(0) & , & 0\leq i\leq h-1-N_{dec}\\ u\left(0\right)\frac{h-1-i}{N_{dec}} & , & h-1-N_{dec}<i\leq h-1. \end{array}\right.$$

where the required number of deceleration samples at the end of the horizon is $N_{dec}=\left\lceil \max\left(\frac{v(0)}{a_{\max}T_{s}},\frac{w(0)}{\alpha_{\max}T_{s}}\right)\right\rceil$ at the current robot velocity $u\left(0\right)={\left[v\left(0\right),\omega \left(0\right)\right]}^{T}$.

Choosing a larger horizon ($h>h_{min}$) also increases safety for moving objects because the motion of the obstacle is predicted early enough to find alternative trajectories that efficiently avoid the collision. However, too large a horizon increases the computational cost and may lead to worse trajectories in free space due to the averaging effect since a longer constrained trajectory does not fit as optimally on the surface of the navigation function.

As a compromise, we choose a larger horizon ($h>h_{min}$) and allow the robot to stop even before the end of the horizon (e.g., at $h_{stop}\leq h$) and keep the remaining number of samples $h-h_{stop}$ still. This effectively makes the horizon variable (with variable stopping time), and since all optimised trajectories have the same number of samples (*h*), their objective functions in Equation (11) are still comparable. The velocity profile in the horizon is then

(10) $$u\left(i\right)=\left\{\begin{array}{ccc} u(0) & , & 0\leq i\leq h_{stop}-1-N_{dec}\\ u\left(0\right)\frac{h_{stop}-1-i}{N_{dec}} & , & h_{stop}-1-N_{dec}<i<h_{stop}\\ {\left[0,0\right]}^{T} & , & h_{stop}\leq i\leq h-1 \end{array}\right.$$

where the candidates for $h_{stop}$ are selected according to the previous optimal curve by evaluating four possibilities $h_{stop}\to h_{stop}+\left\{0,-1,-2,+1\right\}$ that need to be in range $\left(N_{dec}+1\right)\leq h_{stop}\leq h$. Initial value is set to $h_{stop}=h_{min}$.

Optimal control sequence u(0),u(1),…,u(h−1), which minimizes Equation (4), defines the best feasible future trajectory, and its first control action is applied to the robot in the current time. In the next time sample, the procedure repeats.

#### 3.1.3. Convergent Behavior

To ensure convergent behavior of the MPC control, the summands $V\left(s\left(i\right)\right)=N\left(s\left(i\right)\right)+u^{T}\left(i-1\right)Ru\left(i-1\right)$ in the criteria Equation (4) will have to decrease in the horizon. This follows from the convergence constraint in Equation (4)

(11) N(s(i))≥N(s(h)),i=1,…,h.

In the worst case, if all the candidate control actions in Equation (4) result in trajectories that violate the convergence constraint Equation (11), the robot can still choose the optimal trajectory from the previous control step, shifted by one sample. Since the trajectory slows down at the end of the horizon (see Equations (9) and (10)), the robot will start slowing down earlier. This can happen if some dynamic (non-cooperative) objects block its path.

#### 3.1.4. Preventing Conflicts with Dynamic Obstacles

The environment may contain dynamic obstacles that can be treated as cooperative objects (e.g., other robots) and non-cooperative objects (e.g., forklifts operated by humans). Cooperative objects are assumed to have intentions and trajectories known to the robot for at least a prediction horizon *h*. The intentions of non-cooperative objects can be estimated from sensor observations (e.g., laser range scans) of their past movements by estimating their velocities and predicting the most likely trajectories in the horizon.

For a given control u(i) in Equation (4), the robot trajectory s(i) is collision-safe (CS) if it does not collide with any moving object trajectory (static obstacles are already considered in Equation (5)). Let o∈O denote all other moving objects from a set O, and let $s_{o}\left(i\right)={\left[x_{o}\left(i\right),y_{o}\left(i\right),\varphi_{o}\left(i\right)\right]}^{T}$ be the location of an object at horizon prediction time i∈1,…,h

(12) $$\begin{array}{c} CS\Rightarrow ∄o\in O:(||s\left(i\right)-s_{o}\left(i\right)||<d_{safe})\;and\;|\varphi \left(i\right)-\arctan\left(\frac{y_{o}\left(i\right)-y\left(i\right)}{x_{o}\left(i\right)-x\left(i\right)}\right)|<\varphi_{safe}, \end{array}$$

$d_{safe}$ is the required safety distance between the robot and an object *o*, and $\varphi_{safe}$ is the range of angular deviation from the robot’s forward motion that can lead to a collision, arctan(·) is the four-quadrant inverse tangent version. Any robot trajectory that is not collision safe to moving objects is rejected in Equation (4).

The same procedure is followed for all cooperative robots. After one robot determines optimal controls (and trajectory in the horizon), the other robots can adapt by finding collision-proof trajectories to the previous robot. To prevent chattering behavior (where two robots can switch between different optimal controls while avoiding collisions), the selection of optimal controls (trajectories) is done sequentially, which is natural if all robots are controlled from a central computer. If the first robot determines an optimal trajectory that avoids all other robots (taking into account the predicted trajectories of the others), the next robot will adapt by finding collision-safe paths (e.g., swerving to the other side or slowing down). This will automatically lead to consensus since the current predicted trajectories are inherited and known to the others in the same sample. When robots plan and control autonomously, if there is a possibility of collision, they need to only negotiate the order in which they compute their trajectories. Alternatively, they can consider priorities when they are assigned (e.g., for priorities for transportation tasks), as in [10] and [18]. In this way, the first robot computes the optimal trajectory in the prediction horizon, which takes into account the previous trajectories of the others. Additional traffic rules (e.g., swerving to the right for head-on collisions) can also be used [9].

The proposed navigation approach is computationally efficient since the navigation function is precomputed for a static environment, and collisions with dynamic obstacles in the control (Equation (4)) are avoided at runtime.

Note that local minima can still occur (but this is unlikely in practise) if another robot (the second robot or the moving object) approaches another robot (the first robot) exactly from the direction opposite to the negative gradient of the first robot’s navigation function. This also means that the second robot uses a different navigation function, to a different destination whose gradient is in the opposite direction to that of the first robot. When this happens, both robots may slow down as this can be cheaper (according to the MPC control cost (Equation (4)) than driving with the increased values of the navigation functions while avoiding a collision.

In this particular case, it may be a good choice to perform a dynamic replanning of the navigation functions with the detected possible collision position of the other robot. In this way, no slowdown or local minima can occur since the navigation function also considers moving obstacles. This replanning requires additional computation time and should therefore be performed incrementally using the dynamic E* algorithm [25]. Moreover, a relatively fine grid should be chosen (e.g., at most half the robot size) to reduce the discretization error when the predicted collision location is snapped to a grid. Therefore, replanning can only occur if an object is in the collision with the robot in the predicted horizon. Note that the presented MPC approach remains the same if replaned navigation function is used in (Equation (4)), where the objects contained in the navigation function will no longer appear as dynamic collision constraints (Equation (12).

## 4. Optimisation Strategy in MPC Control

In the following, we propose a novel optimization strategy for solving (Equation (4)) that combines optimization with a fixed set of control action candidates and particle swarm optimization.

### 4.1. Fixed Candidate Optimization

To reduce the computational cost, Fixed Candidate Optimization (FCO) is introduced in [36] to solve the MPC problem. Given the current velocities $u_{c}={\left[v_{c},\omega_{c}\right]}^{T}$ (applied to the robot at time $t-T_{s}$), a set of possible discrete accelerations $a_{c}\in \left\{-a_{max},0,a_{max}\right\}$, $\alpha_{c}\in \left\{-\alpha_{max},0,\alpha_{max}\right\}$ is defined to produce a set of 9 candidate velocities for optimization

(13) $$u\left(t\right)=u_{c}+T_{s}{\left[a_{c},\alpha_{c}\right]}^{T}$$

constrained by Equation (7).

The main strengths of the proposed MPC with FCO are the low computational complexity and the generation of near-optimal trajectories with a guaranteed convergence, as shown in [36]. However, the obtained velocity profile contains higher noise due to the coarse set of possible accelerations.

### 4.2. Particle Swarm Optimization

Particle swarm optimization (PSO) uses a stochastic strategy with a swarm of randomly perturbed particles to find a solution. Applying PSO to the MPC problem yields arbitrary velocities u(t) sampled from a continuum and constrained by Equation (7). Each particle *k* is parameterized by a parameter vector $p_{k}={\left[v_{k},\omega_{k}\right]}^{T}$ defining its velocities and an increment vector $\Delta p_{k}$ defining the change in velocities. During MPC optimization, the population of all particles is iteratively updated and validated according to the objective function (4). Each particle keeps track of its parameters and remembers its best previously achieved parameter ${\mathrm{pB}}_{k}$, along with its associated objective function $J_{k}=f\left({\mathrm{pB}}_{k}\right)$, where *f* is the function that is minimized in Equation (4). During optimization, the global best parameter vector of the entire swarm gB is also remembered.

In each sample time, the particles are iteratively updated according to the following rules

(14) $$\begin{array}{c} \Delta p_{k}\leftarrow \gamma \Delta p_{k}+c_{1}\mathrm{rand}\left(0,1\right)\cdot \left({\mathrm{pB}}_{k}-p_{k}\right)+c_{2}\mathrm{rand}\left(0,1\right)\cdot \left(\mathrm{gB}-p_{k}\right)\\ p_{k}\leftarrow p_{k}+\Delta p_{k}, \end{array}$$

where γ>0 is the inertia factor, $c_{1}>0$ is the self-cognitive constant, $c_{2}>0$ is the social constant, and rand(0,1) is a vector of uniformly distributed values in the range [0,1]. At the end of the optimization, the best parameter is applied to the robot u(t)=gB(t).

MPC with PSO produce smoother velocity profiles and can find better solutions since no velocity discretization is used. However, the computational complexity becomes much higher (compared to MPC with FCO) due to multiple required iterations with more particles. Due to the random nature of PSO, both the solution and the convergence of the search are not guaranteed.

### 4.3. Combined Deterministic-Stochastic Optimization

The main idea is to combine FCO and PSO in the so-called combined deterministic-stochastic optimization (CDS) and to exploit the advantages of both algorithms to generate trajectories with a smooth speed profile, with guaranteed convergence and low computational complexity.

CDS is a modified PSO algorithm (shown in Algorithm 1) that executes $K_{F}=9$ fixed particles and $K_{C}$ changing particles in parallel. Fixed particles are initialized by Equation (13) and are not updated during optimization. These fixed particles provide good starting parameters that can be used by other changing particles through gB when iteratively updated through Equation (14). In this way, CDS provides a better (more optimal and smoother) or at least as good a solution as FCO itself. MPC with CDS is guaranteed to converge to the goal in a finite time from any unoccupied location in the environment where the goal is reachable (N(x,y,φ)<∞). The algorithm is computationally efficient since the number of changing particles $K_{C}$ in CDS can be much smaller than in the corresponding PSO.

**Algorithm 1** Combined deterministic-stochastic optimization.**Require:** List of particles k=1,⋯,K where first $k=1,\ldots ,K_{F}$ are fixed particles.   **for** each particle $k=1,\cdots ,K_{F}$
**do**      Initialize $p_{k}$ by Equation (13).   **end for**   **for** each particle $k=K_{F}+1,\cdots ,K$
**do**      Randomly initialize $p_{k}$, $\Delta p_{k}={\left[0,0\right]}^{T}$, ${\mathrm{pB}}_{k}=p_{k}$.   **end for**   $J_{\mathrm{best}}=\infty$, iter=1   **repeat**      **for** each particle k=1,⋯,k
**do**        **if**
$k>K_{F}$ | iter==1
**then**           Compute objective $J_{k}$ by (4) considering ramp down (Equation (9) and add penalty for Equation (7), Equation (12) violation.           Set convergent condition by Equation (11).           **if**
$J_{k}<f\left({\mathrm{pB}}_{k}\right)$
**then**             ${\mathrm{pB}}_{k}=p_{k}$           **end if**           **if**
$f\left({\mathrm{pB}}_{k}\right)<J_{\mathrm{best}}$ & convergent
**then**             $\mathrm{gB}={\mathrm{pB}}_{k}$, $J_{\mathrm{best}}=f\left(\mathrm{gB}\right)$           **end if**        **end if**      **end for**      **for** each particle $k=K_{F}+1,\cdots ,K$
**do**        Update $p_{k}$ and $\Delta p_{k}$ by Equation (14) constrained by Equation (7).      **end for**   **until**
iter≤MAXiter

## 5. Simulation Results

### 5.1. Single Robot Navigation

**Figure 7:**
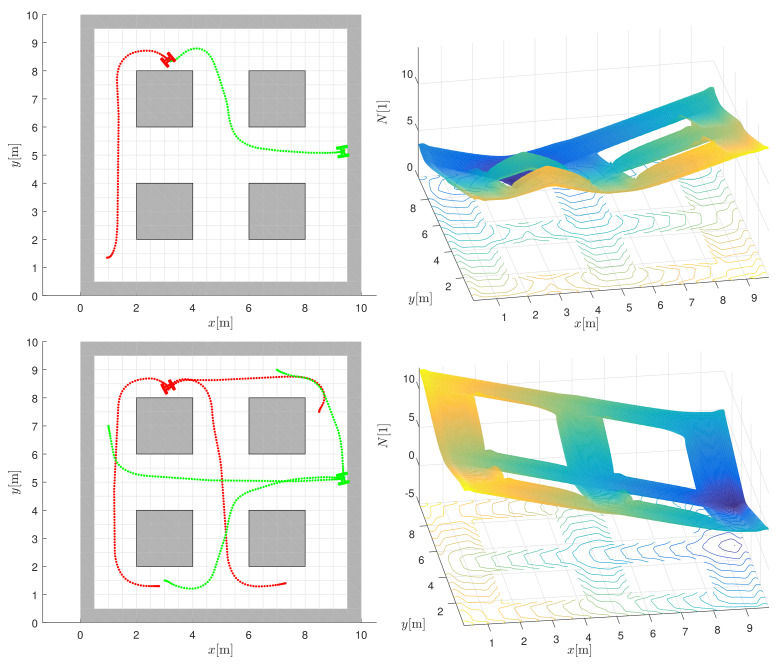
Examples of navigation and control of a single robot in two different destinations defined by the minimum values of the navigation functions in the right column. All red paths are obtained by the navigation function in the top right image, while the green paths are obtained by the navigation function in the bottom right image.

The performance of a single robot in the environment from Figure 1 is first illustrated when the robot needs to transport products or materials from different starting locations to different destinations. A possible scenario could be that the robot has to deliver a semi-finished product to workstation 1 and then transport it to the dropoff location (see top left image in Figure 7).

The navigation functions are computed in advance for known locations, which minimizes the online computational overhead (no online planning is required) and makes the system robust to disturbances during control (e.g., deviations from the original desired path due to errors in robot location, control performance, or dynamic obstacles). Since only a discrete cost map needs to be stored to interpolate the navigation values from it, this is also not memory-intensive (20×20 cost-to-goal for the environment in Figure 1) with the cell size $d_{c}=0.5$ m.

In Figure 7, the desired target is at x=3.1 m, y=8.3 m (e.g., workstation 1 in Figure 1) for red paths. The destination can be safely reached from any location considering the navigation function (the top right image in Figure 7) in the proposed MPC control. For a different desired destination (e.g., drop-off location at x=9.3 m, y=5.1 m in Figure 1), a different navigation function is used for all green paths (the bottom right image in Figure 7). The simulation results are obtained using the following parameters. The interpolation of the navigation function is performed on the grid-based search with a cell size resolution of $d_{c}=0.5$ m. As the interpolation is applied, good navigation and control performance can be obtained even at coarse resolutions. Optimization in MPC is performed using a fixed horizon length h=14 (with deceleration at the end, as shown in Equation (9)) and sample time $T_{s}=0.1$ s, and by considering the constraints on velocities and accelerations $v_{max}=1$ ms${}^{-1}$, $\omega_{max}=6$ s${}^{-1}$, $a_{max}=1$ ms${}^{-2}$, and $\alpha_{max}=6$ s${}^{-2}$.

**Figure 8:**
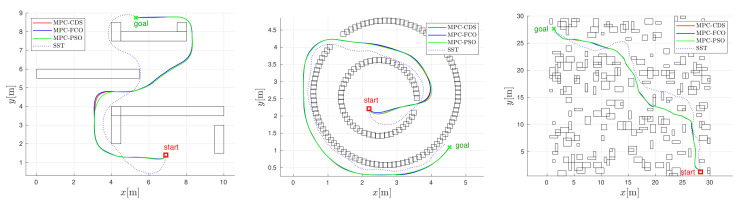
Trajectory comparison for the U-obstacle map (left), the Maze map (middle), the Random-obstacle map (right), the bicubic interpolated navigation, and the SST algorithm.

**Figure 9:**
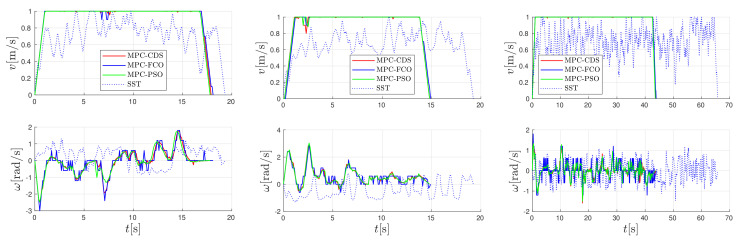
Comparison of velocity profiles for the U-obstacle map (left), the Maze map (middle), and the Random-obstacle map (right).

The performance of model predictive control with the proposed combined deterministic-stochastic optimization (MPC-CDS) is compared with the results of fixed candidate optimization (MPC-FCO) and particle swarm optimization (MPC-PSO). In addition, MPC-based algorithms are compared with the kinodynamic stable sparse RRT planning approach (SST) approach [19]. The results are shown in Figure 8 and Figure 9 for the U-obstacle map, Maze map, and the Random-obstacle map. All MPC trajectories are computed for the bicubic interpolation function and also collected in Table 1. The results are compared in terms of obtained trajectories, velocity profiles, length of trajectory *L*, travel time $t_{goal}$, cumulative navigation $A_{N}$, and normalized computational efficiency $E_{comp}$ (according to MPC-FCO). The computational complexity of MPC-FCO depends on its implementation. In our case, it allows for real-time operation with a refresh rate of at least 50 Hz on a 2.80 GHz Intel dual-core processor with C++ implementation.

The best performance of MPC is obtained by the PSO optimization approach (Figure 8 and Figure 9 and Table 1), where the obtained trajectories are short and fast and the velocities have a smooth profile. However, the computational complexity of MPC-PSO is higher (than MPC-FCO or MPC-CDS) because it uses 25 particles and 20 iterations to optimize each control sample. Similar performance in terms of trajectory length and travel time is obtained with MPC-CDS, which uses nine fixed particles and only two changing particles. The results of CDS are a compromise between the quality of trajectories generated by PSO and the computational complexity of FCO. CDS produces smoother velocity profiles than FCO and requires much less computational effort than PSO. SST produces similarly long trajectories, sometimes shorter since it does not take into account safety costs around the obstacles, but with much slower velocity profiles due to the randomness of velocity selection during the search process. Unlike the MPC-based algorithms, the SST algorithm computes the entire trajectory to the goal before the robot begins execution.

### 5.2. Multiple Robot Coordinated Navigation

**Figure 10:**
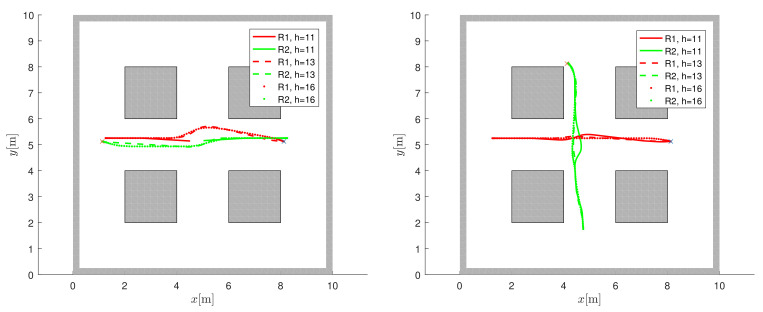
Analysis of horizon length in frontal collision avoidance (left) and cross collision avoidance (right). The target locations are indicated by a cross. A larger prediction horizon can find better trajectories than the minimum horizon $h_{min}=11$.

Analysis of the selection of the horizon length in MPC performance is first explained. The minimum horizon length $h_{min}$ (Equation (8)) is sufficient for navigation in static environment (obstacles mapped or unknown in the navigation function), but it may not be good enough for moving obstacles. For moving obstacles and $h>h_{min}$, safety and navigation performance increases because collision threats can be predicted early enough so that better avoidance routes can be found. The analysis of the varying horizon length (where the moment of deceleration can also occur before the end of the horizon, as defined in Equation (10)) for the navigation of two robots approaching a head-on collision and a cross collision ([9]) is shown in Figure 10 and Table 2. In a head-on collision (left image in Figure 10), the robots stop to avoid collision when the minimum horizon $h=h_{min}=11$ is chosen, while the robots can safely navigate to the target for $h>h_{min}$. This scenario is more difficult than the cross-collision (right part of Figure 10) since the other robots move in the opposite direction of the negative gradient of the navigation function. A larger horizon can provide better collision avoidance but increases the computational cost of navigation ($E_{comp}$ increases in Table 2). $E_{comp}$ is the normalized computational load corresponding to the computational time at $h=h_{min}$ (where in the first line the value $E_{comp}=1$ is normalized by the computational time of the ignored collision, since the robots do not reach the goals). A larger horizon can slightly reduce both the distance traveled (joint distance ∑L in Table 2) and the travel time (joint travel time ∑T in Table 2), which means that the robots do not need to wait or slow down to avoid a collision. Note that the improvement in travel time and distance is relatively small compared to the increased computational cost. Therefore, the main reason for increasing the horizon is safety and collision avoidance performance.

**Figure 11:**
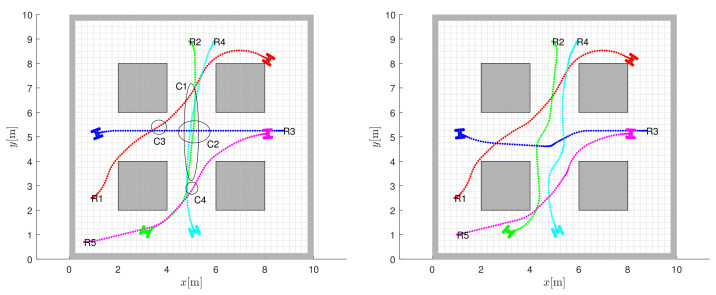
Coordinated collision avoidance. Obtained robot path without using collision avoidance in MPC (where robots drive over each other), with collision cases marked by ellipses (left image). Collision avoidance with prediction horizon h=20 finds safe routes with similar travel times (right image).

Some other examples of coordinated navigation and control of multiple robots can be found in Figure 11, where the navigation results are shown without collision avoidance (left images, where the robots drive over each other) and with coordinated collision avoidance navigation (right images). The starting position of the *i*-th robot is marked with Ri, and its target position coincides with the final robot position. The occurrence of collisions is marked (left image in Figure 11) by ellipses Ci, where C1 is the first collision between robots 2 and 4; C2 between robots 2, 3 and 4; C3 between robots 1 and 3; and C4 between robots 4 and 5. The controller with coordinated predictive collision avoidance (right image in Figure 11) successfully avoids all collisions.

**Figure 12:**
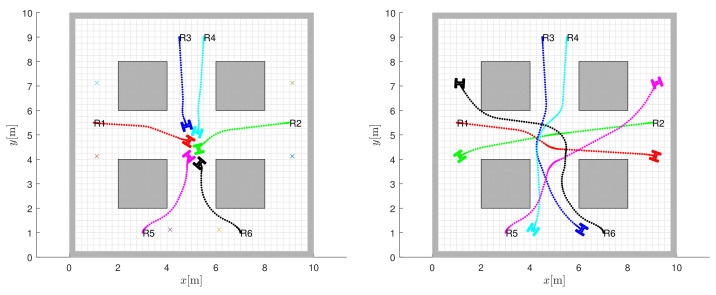
The obtained control with avoidance and prediction horizon h=15 cannot steer the robots towards the destinations since the robots stop safely to prevent collision (left image). Increasing the horizon to h=25 leads to safe trajectories towards the target locations (right image).

Coordinated collision avoidance for symmetric initial locations and congested traffic in the centre of the map is shown in Figure 12. The obtained control with avoidance and prediction horizon h=15 fails to navigate the robots to the destinations as the robots stop safely to avoid collisions (Figure 12, left image). Increasing the horizon to h=25 results in safe trajectories to the targets (Figure 12, right image).

### 5.3. Experiments

**Figure 13:**
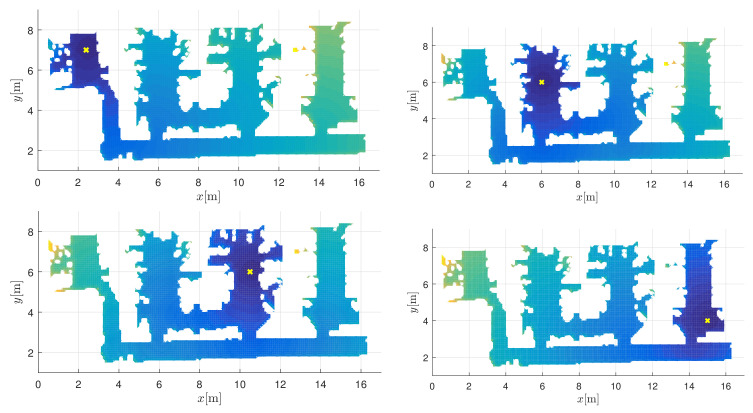
Four interpolated potential fields are used in the navigation functions $N_{i}\left(y,x,\varphi \right)$ (i∈1,…,4) to find one of the desired target positions $G_{N1}={\left[2.4,7\right]}^{T}$, $G_{N2}={\left[6,6\right]}^{T}$, $G_{N3}={\left[10.5,6\right]}^{T}$, and $G_{N4}={\left[15,4\right]}^{T}$ from any initial position. The targets are located at the lowest value of the potential field (darkest region) and are marked by a cross.

Navigation is performed also in the real map shown in Figures 15 and 16 (floor plan of our laboratory). The map is an occupancy grid with 10 cm resolution created with the Sick LMS200 laser range finder. Four target locations $G_{N1}={\left[2.4,7\right]}^{T}$, $G_{N2}={\left[6,6\right]}^{T}$, $G_{N3}={\left[10.5,6\right]}^{T}$, $G_{N4}={\left[15,4\right]}^{T}$ (e.g., locations of workstations) are defined on the map. A robot can reach a desired goal through MPC control (Equation (4)) by following the navigation function (Equation (3)), which consists of an appropriate interpolated potential field. Figure 13 shows potential fields for the defined targets.

**Figure 14:**
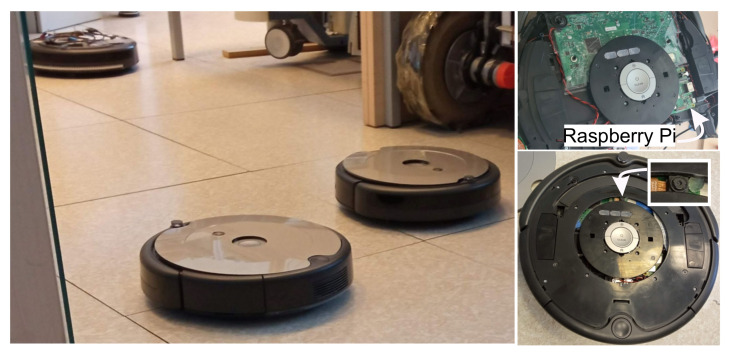
Roomba robots used to simulate transportation tasks in the laboratory layout from Figure 15 and Figure 16. View of the robots (left) and closer robot view with integrated Raspberry Pi and camera (right).

In experiments, Roomba cleaning robots (Figure 14) are used to simulate transportation tasks between desired workstations. For localization, a camera is used to detect Aruco markers on the ceiling placed at known locations. The robots are controlled by a built-in Raspberry Pi, which sends velocity commands with an update frequency of 10 Hz (Ts=0.1 s).

During navigation, velocities and accelerations are constrained by $v_{max}=0.45$ ms${}^{-1}$, $\omega_{max}=3$ s${}^{-1}$, $a_{max}=0.5$ ms${}^{-2}$, and $\alpha_{max}=3$ s${}^{-2}$. To predict collision hazards with other robots, the horizon $h=20>h_{min}$ ($h_{min}=11$ according to Equation (8)) is chosen and safety distance and angle are set to $d_{safe}=0.35$ m and $\phi_{safe}=\pi /2$.

**Figure 15:**
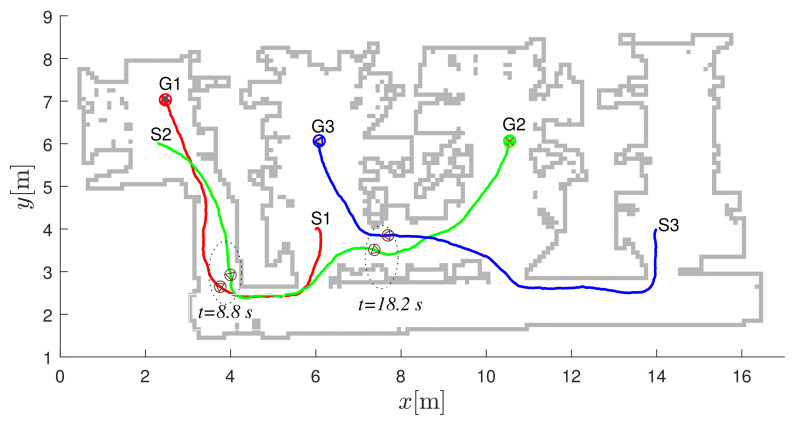
Robot paths in the laboratory layout. The start location of the *i*-th robot is denoted by $S_{i}$ and the destination locations by $G_{i}$. Safe navigation during collision avoidance is represented by dark gray circles belonging to passing robots at the same time (the left pair of circles belongs to time t=8.8 s and the right pair to time t=18.2 s).

In Figure 15, the first robot uses the first navigation function (the upper left image in Figure 13) to get to the destination $G_{1}=G_{N1}$. Similarly, the destination for the second robot is reached by the third navigation function ($G_{2}=G_{N3}$) and the destination for the third robot is reached by the second navigation function ($G_{3}=G_{N2}$).

**Figure 16:**
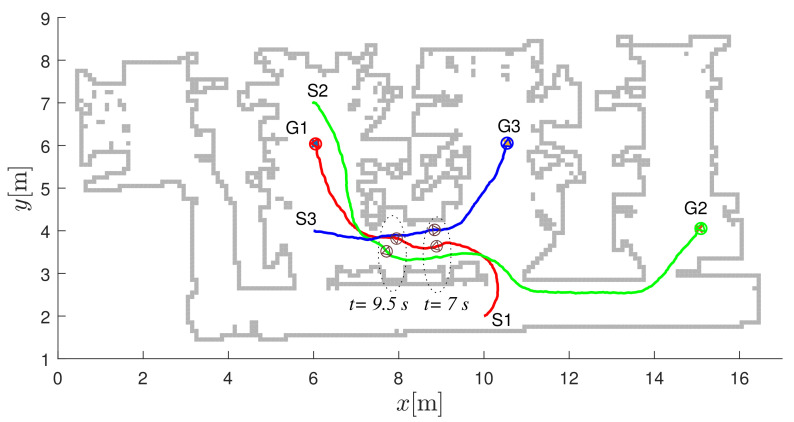
Robot paths in the laboratory layout. The start location of the *i*-th robot is denoted by $S_{i}$ and the destination locations by $G_{i}$. Safe navigation during collision avoidance is represented by dark gray circles belonging to the robots passing simultaneously (the right pair of circles belongs to time t=7 s and the left pair to time t=9.5 s).

In Figure 16, the first robot uses the second navigation function (the upper left image in Figure 13) to reach the destination $G_{1}=G_{N2}$. Similarly, the destination for the second robot is reached by the fourth navigation function ($G_{2}=G_{N4}$) and the destination for the third robot is reached by the third navigation function ($G_{3}=G_{N3}$).

## 6. Discussion

From simulations and experiments, the proposed interpolated navigation function combined with MPC computes collision-safe paths for a single vehicle that are near-optimal considering static obstacles. Moreover, the completeness of the system is guaranteed since the potential field does not contain local minima. The approach assumes that the global information about the system layout is known and static. This allows a discrete potential function (e.g., a distance-to-goal cost map) to be computed in advance and bicubic interpolation to be performed only at runtime to obtain a computationally efficient continuous estimate of the potential field values and their negative gradients. In manufacturing or similar applications, vehicles need to deliver cargo between several defined destinations. For each destination, a suitable navigation function can be precomputed, which increases the computational efficiency.

Good trajectories are also obtained when avoiding collisions with multiple robots. Other robots or moving objects are only considered locally within the prediction horizon. Choosing a minimum horizon ($h_{min}$) ensures that the robot navigates safely while moving past other robots and, in the worst case, stops to avoid a collision. Extending the prediction horizon (e.g., to $2h_{min}$ or more) allows the robots to navigate safely without unnecessary emergency stops to prevent collision. Since local information is considered, optimality is not guaranteed, although good results are obtained in practice. The robot could navigate into a narrow corridor (following the negative gradient) based on the static navigation function, although there is not enough space to avoid collision with another vehicle. MPC will try to follow the direction that reduces the potential while searching among the possible trajectories to avoid collision with another approaching vehicle. In the worst case, if there is not enough free space, the robots would safely stop before a collision occurs.

It would also be possible to globally take into account information about other moving objects and re-create the navigation function. This would require dynamic replanning of the discrete potential field, which is computationally more challenging. However, since the future positions of other objects are not known in advance (they can only be predicted within the sensor’s field of view), the navigation function would need to be modified early enough for the robot to safely follow the modified negative gradient of the re-planned potential, which would prevent a collision. The same requirements apply to the length of the prediction horizon as for the static potential field (the horizon must be long enough according to $h_{min}$). Additionally, the resolution of the discrete grid would need to be fine (much smaller than the size of the robot) to allow for more accurate updating for detected moving obstacles, which is important for narrow passages. A finer resolution of the grid would further increase the computational complexity.

## 7. Conclusions

In this work, we proposed a novel navigation function obtained from a discrete graph search and smoothed by bicubic interpolation. The navigation function has no local minima and decreases monotonically in the direction of a target, allowing a mobile robot to safely navigate from an arbitrary initial configuration to a desired target. For environments where a set of desired targets is known and fixed, such as on the shop floor or in a warehouse, the appropriate navigation functions can be precomputed. This allows for computationally efficient navigation with rather modest memory requirements. The navigation function is coupled with model predictive control (MPC), which extends navigation to multiple robots and introduces variable horizon and combined stochastic and deterministic search in the optimization to improve performance. Coordination of multiple vehicles is solved locally in MPC as a constrained optimization problem where the cooperating vehicles must share their trajectories in the horizon, while for other objects the trajectories must be estimated from observations. The applicability of the proposed solutions is illustrated by several simulations and experiments. In the future, we will explore alternative approaches for interpolating navigation function. In addition, coordination overhead could be reduced by introducing traffic guidelines and a one-way option in the navigation function, which would improve performance and reduce coordination overhead in narrow corridor areas.

## Figures and Tables

**Table 1:** Algorithms validation on three different maps.

	Alg.	*L* [m]	$t_{\mathrm{goal}}$ [s]	$A_{N}$ [1]	$E_{\mathrm{comp}}$ [1]
**U map**	MPC-CDS	16.88	17.90	230.39	1.61
	MPC-FCO	16.95	18.10	230.84	1.00
	MPC-PSO	16.79	17.80	228.68	13.11
	SST	13.82	19.18	n/a	n/a
**Maze map**	MPC-CDS	13.63	14.80	111.30	1.73
	MPC-FCO	13.64	14.90	111.66	1.00
	MPC-PSO	13.65	14.80	111.73	13.63
	SST	12.87	19.09	n/a	n/a
**Rnd map**	MPC-CDS	42.69	43.70	1090.24	2.90
	MPC-FCO	42.78	43.90	1092.28	1.00
	MPC-PSO	42.59	43.60	1091.18	28.01
	SST	46.07	65.59	n/a	n/a

**Table 2:** Performance at variable horizon.

	$h/h_{\min}$	∑L [m]	∑T [s]	$E_{\mathrm{comp}}$ [1]
**head-on**	11/11	/	/	1.00
	16/11	14.27	16.40	1.46
	22/11	14.26	16.40	2.05
**cross**	11/11	13.48	16.20	1.00
	16/11	13.38	15.90	1.63
	22/11	13.33	15.80	2.27

## Data Availability

Not applicable.
